# Male human papillomavirus infection post-kidney transplant: an overlooked disease

**DOI:** 10.1186/2047-1440-1-21

**Published:** 2012-11-16

**Authors:** Oksana Genzer, Suzanne E El-Sayegh, Morton J Kleiner, Mario R Castellanos

**Affiliations:** 1 Division of Research, Department of Medicine, Staten Island University Hospital, 475 Seaview Ave. Staten Island, New York, 10305, USA; 2 Division of Nephrology, Department of Medicine, Staten Island University Hospital, 475 Seaview Ave. Staten Island, New York, 10305, USA; 3 Division of Medical Women's Health, Department of Medicine, Staten Island University Hospital, 475 Seaview Ave. Staten Island, New York, 10305, USA

**Keywords:** Human papilloma virus, Post-kidney transplantation, Immunosuppression

## Abstract

While immunosuppressive regimens improve the overall survival of renal transplant recipients, they also contribute to the long-term complications of post-transplant malignancies. Chronic immune suppression in renal transplant recipients (RTR) increases the risk of viral-associated cancers. In male RTR, human papillomavirus (HPV) is implicated in the development of penile, anal, oropharyngeal, and non-melanoma skin carcinomas. Despite the significance of this virus in RTR, there is an overall deficiency in the understanding of the natural history of HPV infection in male RTR. In the next 20 years, it is believed that cancers will be the leading cause of death in kidney transplant recipients. HPV-associated carcinomas are of particular interest since they are sexually transmitted and in theory may be preventable diseases. This commentary highlights some of the progress made in understanding how HPV is transmitted amongst couples in the general population. It also summarizes the current knowledge of HPV infection in male RTR and describes the deficiencies in published medical literature.

## Commentary

Renal transplant recipients (RTR) have an increased incidence of human papillomavirus (HPV)-related malignancies, however, studies on the natural history of HPV infection are lacking especially in male RTR. While immunosuppressive regimens improve the overall survival of renal transplant recipients, they also contribute to the long-term complications of post-transplant malignancies [1-6]. In males, oncogenic HPV types are associated with penile, anal, oropharyngeal, and non-melanoma skin cancers [1]. Of significance for male RTRs is that these HPV-associated cancers are also known to be significantly increased post kidney transplantation [6-9]. Ongoing research is being done to examine the molecular mechanism and risk factors associated for HPV carcinogenesis, since epidemiologic studies clearly show an increased risk of HPV-associated cancers post transplantation. Despite this fact, there is limited knowledge on the immunologic alterations post-transplantation that make RTR susceptible to HPV carcinogenesis in males. Understanding how RTR acquire HPV infection at specific organ sites is important since it may have implications for prevention strategies via safe sex practices, screening, and lifestyle modifications post kidney transplant. While all solid organ transplant patients are at risk for HPV-associated malignancies [10] because they share many commonalities, there were differences with regard to patient demographics, co-morbidities and more importantly immunosuppressive regimens used. As a result, examining HPV infection post transplantation requires a comprehensive investigation specific to each individual organ transplant group to better understand the natural history, pathogenesis, and the immunologic microenvironment that makes these patients susceptible to HPV carcinogenesis. Specifically targeting research in the renal transplant population is important because as it is known they are the most common transplantation group. In 2010, out of 28,663 organ transplants that were performed in the US, 16,899 patients received kidney transplants compared to 6,291 liver transplants, 2,333 heart transplants, and 1,770 lung transplants [11]. In addition, after any kind of solid organ transplant, there is an increase in chronic renal failure which will eventually require a renal transplant or hemodialysis. Centers of excellence that care for and monitor large cohorts of patients post kidney transplantation can led this effort and conduct much needed research in understanding the biology of HPV infection and the associated cancers in males RTR.

Our group attempted to establish the prevalence of genital HPV infection in males post kidney transplantation by conducting a systematic review of the published medical literature in the Medline database. Our inclusion criteria consisted of studies that examined HPV by *in-situ* hybridization, PCR, or histopathology. We excluded case reports and studies that evaluated men that had sex with men or anal HPV infection because while it is important to look at all groups of men in the RTR population, heterosexual men have been extremely under-represented in studies, yet they make up the majority of male RTR. Through our search, only 19 citations were identified examining anogenital infection in male RTR; however, they were excluded based on our criteria. To our surprise, there are no published studies that have been done reporting the prevalence of genital HPV in heterosexual male RTR, despite the fact that thousands of men have undergone kidney transplantation.

In addition to urogenital malignancies in RTR, HPV infection has been implicated by some investigators to be associated with having an increased risk of squamous cell carcinoma (SCC) of the skin [11-13]. Unlike mucosal HPV infections in which transforming oncogenes HPV E6 and E7 can causes cancer when viral replication is disrupted, some cutaneous HPV infections are suspected to induce skin keratinocyte transformation via different mechanisms. Human paillomaviruses are categorized in five genera based on DNA sequences. The α group is the most common and includes high-risk HPV (types 16 and 18). The β-HPV genus which produce cutaneous infection have been reported to attenuate the harmful effects of sun exposure [11,12]. In a study by Wallace *et al.*[11], investigators show that β-HPV E6 gene expression along with UVB promoted p300 degradation, this ultimately resulted in increased UVB-induced DNA damage, therefore increased risk of cancer. Furthermore, in another study by Proby *et al.*[14], investigators reported on 210 organ transplant recipients with previous SCC and 394 controls without skin cancer. The presence of 25 beta HPV types was examined for viral DNA and serum antibodies response for the 15 most prevalent beta HPV types. In this study authors found a significant association between SCC when patients had a concordant detection of both antibodies and DNA for at least one beta HPV type present. Though some good evidence exists and has been reviewed [14], studies have not always been consistent in establishing HPV as a cause of SCC of the skin in RTR. Cutaneous HPV infection in the immunocompetent and immunocompromised host both have a high prevalence of at least 90%, yet organ transplant recipients have a 100-fold increase in SCC [1]. Clearly, post transplantation skin carcinogenesis is increased for reasons unknown. Solar as well as environmental factors contribute; cutaneous HPV infection may be a co-factor in some patients [15,16].

When looking at screening tests for HPV in men, the options are limited. Unlike women where established commercial tests are available, HPV testing in men in the clinical setting is not done and published reports in the general population use only the research methods. This is another factor which has limited research for studying HPV infection in men. In women, a localized sample of cervical mucosa is easily collected to test for HPV DNA, while in men the cornified epithelium of the penis and scrotum does not always provide a reliable collection of relevant cells [17]. Therefore, the variable prevalence rates that have been reported in studies that examine HPV infection in men in the general population may be due to inaccurate testing estimates and not true infection rates among populations studied. Furthermore, men are known to harbor HPV infection in multiple sites, including the perineum. Therefore, determining that a male patient is HPV-negative is more complex than in a female patient. Diagnostic tests and procedures need to be established to standardize the collection of samples and detection of HPV DNA in men. This would improve research studies that investigate the natural history of this virus in men.

In the next 20 years, it is believed that cancer will be the leading cause of death post transplantation [9]. HPV-associated cancers of the urogenital tract are of particular interest, since they are sexually transmitted and in theory may be preventable diseases. It is therefore important to look at potentially modifiable risk factors to reduce cancer risk. Sexual practices post transplant can increase the risk of HPV exposure. To date, little is known about HPV post transplantation in heterosexual men. Transplantation specialists and physicians that care for these patients have the opportunity to lead an effort to conduct comprehensive research to study HPV infection. Meanwhile, physician awareness of this disease can lead to better doctor-patient communication to discuss sexual behavior and choices post-kidney transplantation. Counseling patients on ‘safe’ sex practices (latex condom use) or abstinence, mutual sexual monogamy, and discussing patient and partner’s sexual history post transplantation may significantly reduce the incidence of HPV-related malignancies. This is an approach for cancer risk reduction that is practical, cost-effective, and achievable.

## Competing interests

The authors declare that they have no competing interests.

## Authors’ contributions

All authors participated in writing, reading, editing, and approving the final manuscript.
